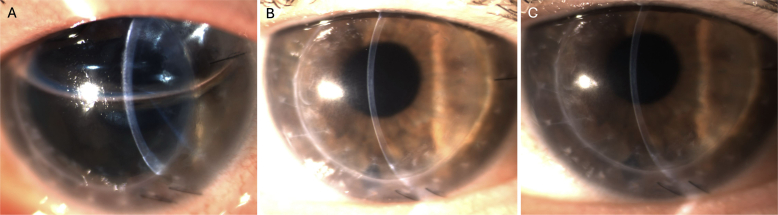# Descemet's membrane endothelial keratoplasty with scraping without descemetorhexis (DMEK-SWD) in a failed penetrating keratoplasty

**DOI:** 10.1016/j.ajoc.2025.102499

**Published:** 2025-12-09

**Authors:** Alexander Wallace, Mohit Parekh, Sabrina Vaccaro, Francesco Semeraro, Vito Romano

**Affiliations:** a Manchester University NHS Foundation Trust, Manchester, UK; b Schepens Eye Research Institute, Massachusetts Eye and Ear, Department of Ophthalmology, Harvard Medical School, Boston, MA, USA; c Department of Molecular and Translational Medicine, University of Brescia, Italy; d Eye Unit, Department of Medical and Surgical Specialties, Radiological Sciences, and Public Health, University of Brescia, Viale Europa 15, 25123, Brescia, Italy

**Keywords:** Cornea, Surgery, Endothelium, DMEK, PK, Endothelial decompensation

## Abstract

**Purpose:**

To introduce a novel treatment for a secondary failed penetrating keratoplasty (PK), termed Descemet's Membrane Endothelial Keratoplasty with endothelial scraping and without descemetorhexis (DMEK-SWD), through the presentation of two clinical cases.

**Observations:**

In this prospective, single-center case series, two patients with previous PK for keratoconus developed endothelial decompensation, presenting with decreased visual acuity (Patient A: counting fingers; Patient B: 0.8 logMAR) and increased central corneal thickness (A: 735 μm; B: 653 μm). DMEK-SWD was performed using a 7.5-mm cell scraper through the temporal port to mechanically remove the host endothelium. A pre-stripped 7.5-mm DMEK graft was inserted using a standardized no-touch technique. Postoperative management included subconjunctival injections of gentamycin and dexamethasone. Graft attachment was maintained in both cases, although Patient B required single rebubbling procedure.

Postoperative graft endothelial cell density (ECD), measured from confocal imaging (HRT3-RCM Cell Count), was 1,125 cells/mm^2^ at 12 months in Patient A and 1,544 cells/mm^2^ at 8 months in Patient B. At final follow-up (A: 18 months; B: 8 months), both patients showed improved best-corrected visual acuity (A: 0.2 logMAR; B: 0 logMAR) and reduced CCT (A: 496 μm; B: 505 μm).

**Conclusions and importance:**

This pilot study demonstrates the feasibility of DMEK-SWD in treating failed PK, with a mean graft clearance time of 1.5 weeks for the two cases analyzed. The technique may represent a promising alternative to repeat full-thickness transplantation and supports further investigation in larger cohorts. Given the very small sample size and the incomplete availability of endothelial cell count data, these findings should be regarded as preliminary.

## 1 Introduction

Late secondary endothelial failure is a leading indication for reintervention after penetrating keratoplasty (PK).^1^ Descemet's Membrane Endothelial Keratoplasty (DMEK) has been established as an effective treatment for PK endothelial failure (DMEK-on-PK) over PK regrafting, given lower rejection risk, better vision, and fewer suture-related complications.^2^, ^3^, ^4^ Additionally, DMEK achieves superior outcomes compared to other endothelial keratoplasty (EK) procedures, such as Descemet stripping automated endothelial keratoplasty (DSAEK) (DSAEK-on-PK), in terms of recovery time and vision.^5^^,^^6^ However, detachment and rebubbling rates with DMEK-on-PK remains a concern.^4^

Technical factors likely contribute. In failed PK, corneal haze impairs visualization during descemetorhexis, increasing the risks of incomplete DM removal and DM-tag formation, which can create an uneven bed predisposing to graft malapposition, decentration, and detachment.^2^^,^^7^^,^^8^

Under normal conditions, descemetorhexis creates a smooth surface for the DMEK adherence. However, during DMEK-on-PK, descemetorhexis may not be mandatory due to the absence of guttae. This is an important consideration, as poor visualization during the procedure can lead to an incomplete descemetorhexis, resulting in an uneven surface that may compromise the DMEK graft. This has led to the proposal of DMEK without descemetorhexis (DMEK-WD), where the DMEK is placed directly in contact with the dysfunctional endothelial cells. Beyond initial case reports, more recent work, including a larger series and additional accounts of non-stripping endothelial keratoplasty, has reported encouraging anatomic and visual outcomes.^7^^,^^9^, ^10^, ^11^

We believe there are further benefits to removing the recipient's dysfunctional endothelial cells via cell scraping. Ex vivo data from our study support the feasibility and biological plausibility of this approach: selective cell scraping with DM left intact resulted in faster centripetal migration and wound closure than DM peeling, and adjunct ROCK-inhibitor exposure further accelerated closure with earlier re-establishment of endothelial phenotype and junctional markers.^12^ Creating this “cell-off/DM-on” interface permits the graft's DM to have direct contact with the recipient's intact DM, improving adherence and attachment.^8^ Furthermore, eliminating these cells may theoretically reduce the risk of graft rejection and limit interference from the dysfunctional host endothelium, which can impact the graft's performance. Studies also indicate that removing dysfunctional endothelial cells promotes the proliferation and migration of peripheral functional cells to the centre of the cornea, aiding in the upkeep of corneal health.^13^^,^^14^

This paper presents two cases of DMEK with endothelial scraping and without descemetorhexis (DMEK-SWD) under a failed PK and investigates the surgical outcomes.

## 2 Methodology

The DMEK graft tissue was stripped by a corneal surgeon using a previously described DMEK stripping technique^15^^,^^16^ and preloaded into a device with the endothelium facing inwards.^17^

The surgery was performed by a corneal consultant (VR). Briefly, side ports were created for the ophthalmic viscosurgical devices. Via the temporal port, the cell scraper device (Janach srl.) was inserted into the anterior chamber, and the endothelium was scraped in a linear pattern along the circumference, with a diameter of 7.5 mm. The 7.5-mm diameter was selected to correspond to the size of the donor graft, ensuring that all dysfunctional endothelial cells within the graft area were removed while minimizing unnecessary trauma to the peripheral host tissue. No intraoperative OCT was used. Completeness of endothelial scraping and preservation of Descemet's membrane were confirmed under the surgical microscope by direct visualization, aided by 0.25 % trypan blue to highlight any residual cells. A balanced salt solution was then injected into the anterior chamber. The procedure is shown in video 1.

Supplementary video related to this article can be found at https://doi.org/10.1016/j.ajoc.2025.102499

The following is/are the supplementary data related to this article:

Multimedia component 1 Intraoperative sequence of DMEK-SWD. Via the temporal port, the cell scraper device was inserted into the anterior chamber, and the endothelium was scraped first in a linear pattern along the circumference, with a diameter of 7.5 mm. The posterior surface was then stained with 0.25 % trypan blue to verify scraping completeness (blue-stained residual cells vs clear, cell-free DM), followed by balanced salt solution injection. The video then demonstrates DMEK graft loading and actual insertion, controlled unfolding and centration, and anterior chamber air injection. Multimedia component 1

The DMEK graft (7.5mm) was prepared for injection into the anterior chamber as follows. Firstly, the cartridge containing pre-loaded DMEK was mounted on the injector (Viscoject single-use BIO injector, Medicel, Switzerland). Then an anterior chamber maintainer (23-gauge E.Janach srl, Como, Italy) was connected to the posterior end of the injector and pushed into the rubber plug at the rear exit. The other end of the anterior chamber maintainer was connected to a 5 ml non-Luer-lock syringe. The tissue was gently washed with a balanced salt solution (BSS) from the front opening to ensure the removal of preservation media.^18^^,^^19^ Membrane Blue Dual (D.O.R.C., Netherlands) was then injected into the DMEK cartridge from the terminal side to dye the tissue for 3 minutes, and the excess dye was gently washed out using a balanced salt solution before inserting the tissue into the eye. The injector was then introduced into the anterior chamber through a single 2.7 mm 2-step incision. Finally, the graft was advanced into the anterior chamber by applying gentle taps on the plunger of the 5 ml syringe, which provided a slow and controlled flow of BSS through the rubber plug of the injector.

A standardized no-touch technique was used to unfold the graft and place it centrally. The anterior chamber was completely filled with air for approximately 10 minutes and then partially decompressed, leaving an approximately eighty percent air bubble at the end of surgery; no gas was used, and patients were instructed to remain in the supine position for 24 hours. A 10-0 nylon suture was used to close the main corneal wound, and the globe was left at a physiological intraocular pressure. Subconjunctival injections of antibiotic (gentamycin) and steroid (dexamethasone) were administered.

Scheimpflug photography (Pentacam; Oculus Optikgeraete GmbH, Wetzlar, Germany) was used to perform corneal topography. For both cases, the donor corneal graft had a preoperative endothelial cell density of 2,800 cells/mm^2^, as determined by specular microscopy at the eye bank. Postoperative graft ECD was measured on HRT3-RCM images using the built-in Cell Count tool (Heidelberg Retina Tomograph 3 with Rostock Cornea Module), as previously described.^20^

The patient data and imaging were collected from hospital records and securely stored. The pre-operation and follow-up best corrected visual acuity (BCVA), central corneal thickness (CCT), and maximum keratometry (Kmax), were collected, as well as the follow-up contrast sensitivity, time for the graft to clear, and any complications, including rebubbling.

Graft clearance time was defined as the interval from surgery to the first postoperative visit at which the central cornea appeared clinically clear on slit-lamp examination (absence of stromal edema with iris details visible). All assessments were performed by a cornea specialist (V.R.).

## 3 Case A

A 58-year-old woman with a history of PK for advanced keratoconus and prior cataract surgery presented with left-eye corneal decompensation. BCVA was counting fingers, with central haze, CCT 735 μm, Kmax 50.90 D, and a history of IOP elevation managed with topical hypotensives; IOP was controlled at presentation. Baseline parameters are presented in Table 1.

**Table 1:** Baseline characteristic of cases A and B.

Baseline characteristic	Case A	Case B
**Age,** years	58	65
**Gender**	Female	Male
**Previous Surgery**	PK	PK
	Phaco + IOL	Phaco + IOL
	YAG laser capsulotomy	DMEK-SWD
	DMEK-SWD	
**BCVA,** logMAR	CF	0.8
**CCT,** μm	735	653
**Baseline Kmax,** D	50.90	61.2
**Manifest refraction**		
2 weeks		−2.00 −5.00 × 130
1 month		−2.00 −3.50 × 180
8 months		−2.00 −2.50 × 180
18 months	−5.00 × 150	

**Fig. 1:**
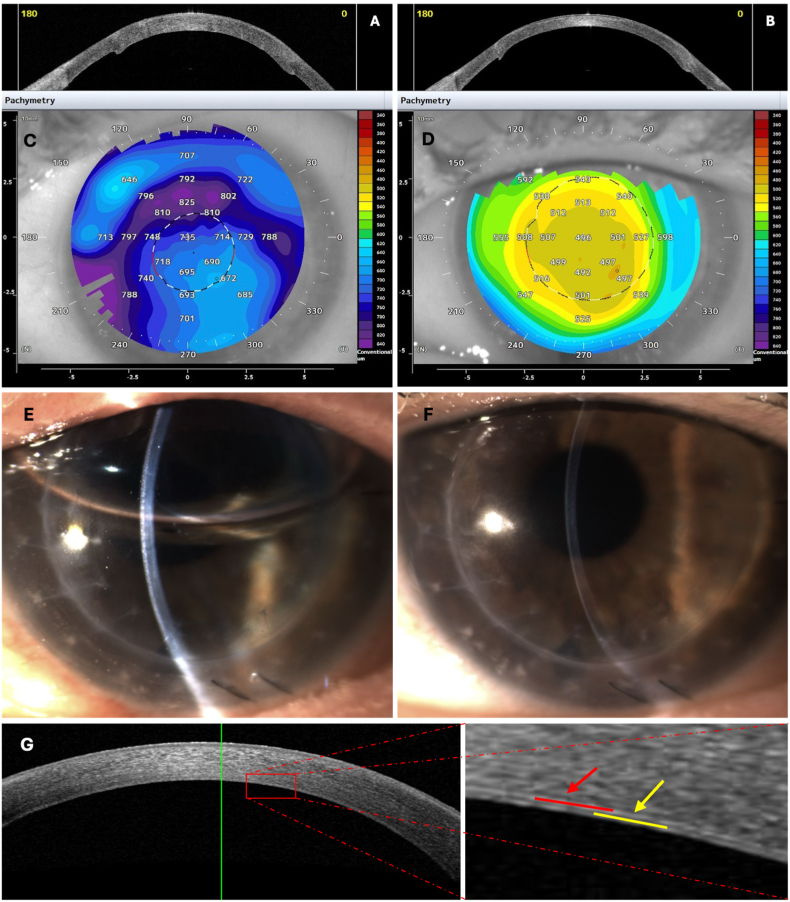
**Case A:** A) AS-OCT before DMEK-SWD, B) 18 months after DMEK-SWD, C) pachymetry before DMEK-SWD (CCT 735μm), D) pachymetry after 18 months DMEK-SWD (CCT 496μm), E) Slit lamp photograph at 1 day after DMEK-SWD surgery, F) slit lamp photography 18 months post-DMEK-SWD revealing a clear graft, G) AS-OCT after DMEK-SWD showing the graft-host interface; red arrows = recipient DM; yellow arrows = graft DM. All AS-OCT panels are 11 mm horizontal scans; scale bar = 1 mm. Anterior segment ocular coherence tomography (AS-OCT), Central Corneal Thickness (CCT), Descemet's Membrane Endothelial Keratoplasty with endothelial scraping and without descemetorhexis (DMEK-SWD), Decemet Membrane (DM).

Left eye DMEK-SWD was performed. The patient maintained a supine position for 24 hours post-surgery. Graft attachment was verified one day after surgery using AS-OCT and slit lamp examination (Fig. 1). Postoperative therapy involved topical dexamethasone/netilmicin eye drops administered six times daily for 14 days, following fluorometholone 0.1 % drops four times daily for four weeks. This was then replaced by prednisolone 1 % four times daily, tapered to once daily at one year. Steroid tapering was not specifically modified due to the prior PK history; the standard protocol routinely used after DMEK in our center was applied, with adjustments only in case of individual postoperative findings such as intraocular pressure elevation. The graft cleared within 2 weeks postoperatively. The graft maintained its centration and transparency, with no need for rebubbling (Fig. 1).

At 12 months, graft ECD was 1,125 cells/mm^2^. At 18 months, left eye refraction showed plano −5.00 × 150, BCVA was 0.2 logMAR, contrast sensitivity measured at 0.90 logCS, Kmax had reduced to 47.77D, and CCT measured 496μm (Fig. 1).

## 4 Case B

A 65-year-old man with prior bilateral PK for keratoconus presented with right-eye endothelial decompensation: BCVA 0.8 logMAR, CCT 653 μm, and Kmax 61.2 D. Baseline parameters are presented in Table 1.

**Fig. 2:**
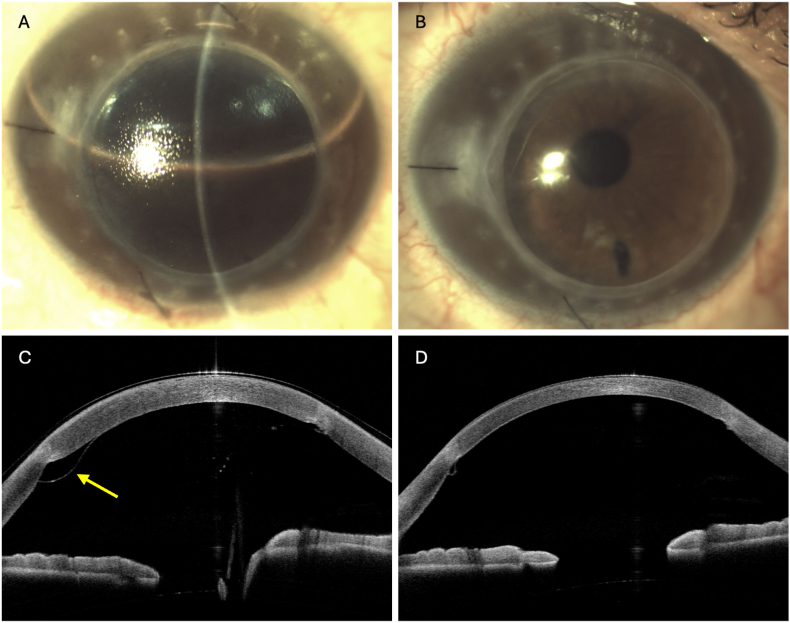
**Case B:** A) Slit lamp photograph after rebubbling, B) slit lamp photography five months post-DMEK-SWD revealing a clear graft, C) AS-OCT showing graft detachment in an area of uneven interface adherence, yellow arrows = graft DM, D) AS-OCT after the rebubbling showing the graft attached. All AS-OCT panels are 11 mm horizontal scans; scale bar = 1 mm. Anterior segment ocular coherence tomography (AS-OCT), Descemet's Membrane Endothelial Keratoplasty with endothelial scraping and without descemetorhexis (DMEK-SWD), Decemet Membrane (DM).

Right-eye DMEK-SWD was performed. Postoperative therapy was administered according to the previously described protocol. Postoperatively, partial graft detachment was noted in an area of uneven interface adherence. In the following days, a progressive detachment occurred, and a rebubbling procedure to reattach the graft was performed (Fig. 2).

Before rebubbling, visual acuity was counting fingers (manifest refraction not feasible due to edema). The graft cleared within 1 week post-rebubbling procedure. BCVA progressively improved following surgery, from 0.5 logMAR, with manifest refraction −2.00 −5.00 × 130 at 2 weeks postoperatively, to 0.3 logMAR at 1 month −2.00 −3.50 × 180, and reached 0 logMAR, −2.00 −2.50 × 180, at 8 months. At eight months, contrast sensitivity was 0.90 logCS, CCT 501 μm, Kmax 58.7 D, and graft ECD 1,544 cells/mm^2^, corresponding to a 44.8 % cell loss. Although the early outcomes were excellent, the follow-up for this case remains relatively short, and the possibility of late failure or rejection cannot be excluded.

IOP remained within normal limits during the follow-up period. No complication occurred after the rebubbling (Fig. 2).

## 5 Discussion

These cases highlight the successful use of DMEK-SWD to treat a failed PK. The objective was to demonstrate the feasibility of scraping the endothelial cells while preserving the DM.

Late endothelial failure is a leading complication of PK, occurring in 13 % of cases after 20 years.^1^ DMEK-on-PK has been established as the preferred treatment over PK regrafting. DMEK-on-PK elicits a reduced immune response than PK regrafting, resulting in a lower risk of rejection and improved graft survival.^2^, ^3^, ^4^ Additionally, it carries a lower risk of complications, such as suture-related infection or bleeding.^2^, ^3^, ^4^

Compared to other endothelial keratoplasty procedures, such as DSAEK, DMEK-on-PK has also demonstrated superior visual outcomes and more rapid recovery.^5^^,^^6^ However, DMEK-on-PK is associated with higher 12-month failure rates (7–37 %) and rebubbling rates (28–56 %) than DSAEK-on-PK.^4^ These higher failure and rebubbling rates are likely due to technical challenges. In cases of PK failure, the cornea becomes hazy, impairing visualization during descemetorhexis.^2^^,^^7^^,^^8^ Consequently, there is a risk of incomplete DM peeling and DM tag formation, leading to an uneven surface for the DMEK to attach, increasing the possibility of detachment.^2^^,^^7^^,^^8^ Furthermore, the DM in failed PK cases can become thinner and friable, making it more difficult to strip, also leading to the possibility of inadvertently opening the graft-host junction while attempting descemetorhexis.^9^

The outcomes of performing DMEK-on-PK without descemetorhexis show relative promise, though this is limited to just a few case series.^7^^,^^9^ However, directly overlying the DMEK graft onto the damaged endothelial cells increases the risk of graft detachment and rejection.^8^

To contextualize DMEK-SWD among related techniques, conventional DMEK-on-PK with descemetorhexis removes recipient DM and dysfunctional endothelium but can be technically challenging in hazy failed-PK corneas, with risks of incomplete peeling, DM tags, interface irregularities, and manipulation near the graft–host junction.^2^, ^3^, ^4^, ^5^, ^6^, ^7^, ^8^, ^9^ In cases of failed PK, DMEK-SWD can easily be performed since there are no guttae. Moreover, during poor visualization, scraping poses less risk than descemetorhexis.^2^^,^^8^

DMEK-WD places the graft over intact DM and overlying dysfunctional endothelium; this avoids stripping when visualization is poor but may leave interference at the interface, and published series remain small and heterogeneous.^7^^,^^9^, ^10^, ^11^ DMEK-SWD preserves the original DM, allowing for DM-to-DM contact. This minimizes the risk of graft rejection and improves graft attachment compared to overlying the DMEK directly onto dysfunctional endothelial cells.^8^ The presence of DM also promotes endothelial healing and reduces corneal fibrosis, contributing to improved visual outcomes.^21^

DMEK-SWD selectively scrapes the host endothelial monolayer while preserving recipient DM, then implants DMEK, creating a “cell-off/DM-on” interface that may promote early adherence and eliminates dysfunctional cells that might otherwise compete or signal adversely. These hypothesized advantages are supported by our ex-vivo observations, in a human cornea model, preserving DM led to faster endothelial migration and earlier defect closure than bare stroma, with further acceleration after short-term ROCK-inhibitor exposure,^12^ and merit evaluation in prospective comparative studies.^8^^,^^12^ Although scraping directly over DM raises a theoretical risk of microtrauma, in our series no intraoperative DM disruptions or postoperative signs of impaired adhesion were observed. Careful, tangential scraping under trypan blue visualization appears to preserve DM integrity; nonetheless, studies with longer follow-up are required to confirm long-term safety.

While DMEK-SWD offers potential advantages by preserving the recipient DM, patient selection remains critical. The technique may not be suitable in cases with significant host DM damage, scarring, or the presence of guttata, where retaining DM could compromise the graft–host interface. In such situations, conventional DMEK-on-PK with descemetorhexis remains the preferred approach.

This study is limited by its retrospective nature and only contains two cases. Additional constraints include elevated intraocular pressure in case A and a relatively short follow-up of eight months in case B. A major limitation is the incomplete ECD dataset, with postoperative graft ECD captured only at limited time points. Moreover, postoperative ECD was derived from HRT3-RCM rather than specular microscopy; while supported in the literature, these modalities are not fully interchangeable across settings. In addition, the follow-up for one case was relatively short, which precludes definitive assessment of long-term graft survival and rejection risk. Future studies should include standardized, longitudinal ECD assessments at predefined intervals to enhance generalizability.

## 6 Conclusions

These two cases demonstrate the feasibility of treating a secondary failure PK with DMEK by scraping the endothelial cells rather than performing descemetorhexis. This approach may minimize rebubbling and failure rates, enhance endothelial cell migration, and improve visual outcomes. While further research is needed to validate this technique, we hope this study will encourage its adoption and future investigation.

## CRediT authorship contribution statement

**Alexander Wallace:** Writing – review & editing, Writing – original draft, Visualization, Validation, Methodology, Data curation. **Mohit Parekh:** Writing – review & editing, Validation, Project administration, Investigation, Data curation, Conceptualization. **Sabrina Vaccaro:** Writing – review & editing, Writing – original draft, Validation, Methodology, Investigation, Data curation. **Francesco Semeraro:** Writing – review & editing, Visualization, Validation, Supervision, Project administration, Conceptualization. **Vito Romano:** Writing – review & editing, Visualization, Validation, Supervision, Project administration, Conceptualization.

## Patient consent

Written informed consent was obtained from the patients for publication.

## Authorship

All authors attest that they meet the current ICMJE criteria for Authorship.

## Funding

No funding or grant support.

## Declaration of competing interest

The authors declare that they have no known competing financial interests or personal relationships that could have appeared to influence the work reported in this paper.

## Figures and Tables

**figs1:**